# Sport and the LGBTIQ+ Community: A South Australian Study

**DOI:** 10.3389/fpsyg.2021.671586

**Published:** 2021-05-05

**Authors:** Murray Drummond, Sam Elliott, Claire Drummond, Ivanka Prichard, Lucy Lewis, Nadia Bevan

**Affiliations:** ^1^Sport, Health, Activity, Performance and Exercise (SHAPE) Research Centre, Flinders University, Bedford Park, SA, Australia; ^2^Caring Futures Institute, Flinders University, Bedford Park, SA, Australia; ^3^School of Social and Political Sciences, Faculty of Arts, Monash University, Clayton, VIC, Australia

**Keywords:** LGBTIQ+, sport, qualitative research, policy and practice, South Australia

## Abstract

This is a paper based on research with the LGBTIQ+ community in South Australia, the likes of which has not been conducted previously in the state. The paper, which utilized both quantitative (*n* = 148) and qualitative (*n* = 31) research methods identifies the key issues that the LGBTIQ+ community face with respect to sporting involvement. There were a range of themes that emerged in relation to a variety of topics including homophobia, sexism and gender discrimination, gender roles and gender stereotypes. This paper provides data and discussion around this important part of the research, which has implications pertaining to sporting organizations and the LGBTIQ+ community.

## Introduction

Sport has been historically constructed and reconstructed as a male-oriented domain and a site in which hegemonic masculinity has been the dominant ideology, particularly within institutionalized sport (Messner, 1992; Connell, 1995; Fink, 2008, 2016; Anderson, 2011; Piedra et al., 2017). While sports remain closely aligned to hegemonic masculinity (English, 2017) there is a shifting culture emerging toward acceptance of Lesbian, Gay, Bisexual, Transgender, Intersex and Queer (LGBTIQ+) people within society; sport being a dominant area of participation for the LGBTIQ+ community (Kauer and Krane, 2006; Ensign et al., 2011; Anderson et al., 2012; Oswalt, and Vargas., 2013; Cleland, 2014; Channon and Matthews, 2015; Piedra et al., 2017).

Research on Lesbian, Gay, Bisexual, Transgender and Queer/Questioning + sport experiences largely emerge from elite level, college (Eng, 2008; Anderson et al., 2016) and professional settings (Billings et al., 2015). However, there has been less research (individually or collectively) in community settings (Drummond et al., 2018). Indeed, Baiocco et al. (2019) claims that there may be specific homophobia that occurs within sport related contexts. In Australia, recent research on gender, sexism and homophobia in sport indicates that homophobia and sexism are significant stressors for LGBTIQ+ people within community sport (Symons et al., 2017). Suggestions for advancing the field include engaging stakeholders who have previously been overlooked in discussions surrounding LGBTIQ+ involvement in community sport (Trussell et al., 2018).

Anderson, has undertaken extensive research in gay men in sport (many articles current and dating back to 2002). He has also published research related to lesbians in sport (see Anderson and Bullingham, 2015). According to Anderson and McCormack (2016), p.2) homohysteria is defined as the fear of being socially perceived as gay. Additionally, Piedra et al. (2017) defines the concept of homohysteria as:

*Engaging in actions intended to distance oneself from the suspicion of being gay, Anderson notes that among men, homohysteria is typically manifested by fleeing from feminized behavior, including physical contact with other men or showing signs of affection and emotion, while also maintaining homophobic discourse. Homohysteria helps to explain how gendered patterns of behavior—both within and outside of sport settings—play out in relation to shifting levels of societal homophobia, particularly highlighting how homophobia can affect the behavior of individuals who may not necessarily be homophobic themselves* (P. 1019).

Anderson et al. (2016) state that the incidence of homohysteria and homophobia can decrease over time in society. However, this needs to be led by social, cultural and legislative processes (Piedra et al., 2017), especially as homohysteria can be part of organizational or institutional culture, such as sport (Anderson and Bullingham, 2015). Indeed, findings from various international studies in lesbian, gay and bisexual people in sport show that there are still prevalent issues of homophobia, homohysteria, discrimination, abuse, bullying, othering, stigmatizing and silencing against LGBTIQ+ players, parents, coaches and the wider LGBTIQ+ community in sporting environments (Mattey et al., 2014; Denison and Kitchen, 2015; Lee and Cunningham, 2016; Waldron, 2016; Piedra et al., 2017; Symons et al., 2017; Petty and Trussell, 2018; Trussell et al., 2018). In relation to sporting organizations, Waldron (2016) states:

*The lack of diversity training on issues of gender and sexuality reiterates that gender and sexuality are not valued and important within a sport organization and team.*

Denison and Kitchen (2015) and Symons et al. (2017) are two Australian studies released related to the LGBTIQ+ sporting community. Denison and Kitchen’s study entitled ‘Out on the Fields’ was the first international study on homophobia in sport and included research participants from six Westernized countries; Australia, United States, United Kingdom, Canada, Ireland, New Zealand, in addition to an ‘other’ category capturing multiple other countries (2015). According to participants 80% witnessed or experienced homophobia in sport in Australia, with 85% of gay men and 84% of lesbians personally targeted heard verbal slurs such a ‘dyke’ or ‘faggot’ (Denison and Kitchen, 2015). Youth sport is an area of vulnerability, with 70% of gay people stating that team sport is not safe for gay people, including considerable fear of discrimination from players and officials (Denison and Kitchen, 2015). One per cent of participants in the study stated that LGBTIQ+ people were ‘completely accepted’ in sporting culture, compared to almost 50% stating that LGBTIQ+ people are ‘accepted a little’ or ‘not accepted at all’ (Denison and Kitchen, 2015). Denison and Kitchen’s study (2015) found that in Australia, Rugby Union was the most popular sport for adult gay males. However, as Rugby Union is not as prominent in South Australia as it is in the Eastern States of Australia, mainly NSW and QLD (Rugby.com.au, 2021), there is less certainty as to the type of mainstream and niche sports that have a higher proportion of LGBTIQ+ participants, together with how their needs are being addressed in South Australian sporting organizations.

Symons et al. (2017) examined LGBTIQ+ sexist and homophobic discrimination in sport in Australia, specifically in Victoria. It was found homophobia still is a significant stressor for young people, particularly in educational and sporting settings (Symons et al., 2017). Symons et al. (2017) found that men experience approximately double the level of explicit and implicit homophobia in comparison to women. The study noted that in response to homophobia ‘identity impact’ occurred, that is, the way in which LGBTIQ+ people perceived themselves or, the manner in which they acknowledged or expressed their own sexual identity (Symons et al., 2017). This could lead to feelings of negativity based on their gender or sexuality or perpetuate the notion that diverse sexualities should remain hidden. Homophobia can create feelings that LGBTIQ+ individuals are not ‘OK’ or are ‘less than’ and underpins that LGBTIQ+ people are not welcome in sport, and if wanting to participate, sexualities should remain hidden (Meyer, 2003; Symons et al., 2017). Importantly, Symons et al. (2017) state that:

*When discrimination and abuse is embedded at the organizational level and ‘taken for granted’ the culture of the sport is more difficult to challenge individually. This is especially so for a minority who are rendered largely invisible and lack solidarity due to the effects of heterosexism, stigma and shame. Safety through the ‘closet’ is often a necessity but can also work against direct action for positive change (P. 484).*

This current study is the first South-Australian specific study to identify the sporting sectors influence for LGBTIQ+ participants. It was important to attain sporting participants’ and key stakeholders’ narratives to garner a holistic understanding about the sporting landscape for LGBTIQ+ individuals. The study was also designed to explore elements of potential homophobia and homohysteria in South Australian sport and creating inclusive environments for LGBTIQ+ participants and allies. This study aimed to understand the barriers and the enablers that exist, how these barriers can be minimized and what enablers for LGBTIQ+ people are currently in place that can be enhanced or promoted to other clubs/sports/organizations. This project comprised a crucial step for the South Australia sport industry to recognize and promote inclusive environments to the voices of LGBTIQ+ athletes and support decisions to ‘come-out’ (Barber and Krane, 2007; Fink, 2012; Melton and Cunningham, 2012; Petty and Trussell, 2018).

## The Research

This project utilized a mixed-method methodology to ensure that a comprehensive overview of the sporting climate for the LGBTIQ+ South Australian community was undertaken. It should be noted that this research was conducted with full ethics approval from the Social and Behavioural Research Ethics Committee at the host institution. The research participants all signed consent forms and were given the opportunity to withdraw at any time without prejudice.

### Quantitative

Phase One of the study was a comprehensive, online questionnaire (approximately 15 min), designed to access a diverse range of genders and sexualities, including, albeit not limited to, those who identify as heterosexual, lesbian, gay, bisexual, intersex, transgender, queer, asexual or other, who are current sport participants or ex-sport participants aged 18 years and over. The purpose of this first phase was to attain an all-inclusive perspective on the current landscape of the LGBTIQ+ sporting community in South Australia. The questionnaire included key demographics, and questions related to discrimination, the structure of sporting club’s inclusivity, myths surrounding sexuality in sport, homophobia in sport, and other barriers and enablers of sporting participation for the LGBTIQ+ community. The questions were therefore underpinned by previous research projects used where quantitative measures comprised the primary methodology (e.g., Piedra et al., 2017; Symons et al., 2017), older research in the field (Herek, 1984, 1988), qualitative research projects (White et al., 2020) as well as the expertise of the authors with significant quantitative research experience across a range of large-scale sporting and health-oriented research projects.

### Participant Characteristics

One hundred and eighty-three people commenced the survey; however, 35 participant responses were excluded from analyses due to incomplete data (i.e., they had not completed ≥50% survey). Of the 148 included participants, 93 identified as part of the LGBTIQ+ community and 53 participants identified as heterosexual, and a further two people preferred not to say. From the entire sample, nine participants reported being transgender (6.1%; 7 within the LGBTIQ+ group; 2 within the heterosexual group; 4 male, 1 female, 1 non-binary, 3 other). Of those in the LGBTIQ+ community, participants identified as bisexual (20.9%, *n* = 31; 4 male, 27 female), lesbian (18.9%, *n* = 28), gay (12.8%, *n* = 19; 14 male; 4 female; 1 other), pansexual (4.7%, *n* = 7; 1 male; 4 female; 2 other), other (2.7%, *n* = 4; 2 female, 2 other), queer (2.0%, *n* = 3; 1 female; 2 non-binary), and asexual (0.7%, *n* = 1; 1 male). Of the 93 participants from the LGBTIQ+ community 76.3% (*n* = 71) indicated that they had “come out,” and of these, the majority had come out to friends (97.2%), family (88.7%), spouse/partner (73.2%), sporting club/organization (59.2%) and their workplace (57.7%). For the purposes of this paper, given the small number of participants from some LGBTIQ+ groups, where relevant, descriptive analyses are presented for the whole sample, as well as separately for heterosexual and LGBTIQ+ participant groups. The average age of the sample was 32.0 (*SD* = 11.3) years, with participants in the LGBTIQ+ community being slightly younger (*M* = 29.6 years, *SD* = 9.7) than heterosexual participants (*M* = 36.0 years, *SD* = 12.9). The majority of participants were female (overall: 68.7%, *n* = 101; LGBTIQ+: 71.7%, *n* = 66; heterosexual: 62.3%, *n* = 33). Most of the sample were Caucasian (82.3%, *n* = 121) and currently employed (70.9%, *n* = 105).

#### Involvement in Organized Sport

Table 1 presents participant involvement in organized sport, whether participants had ever dropped out of sport, and whether there were any sports that participants did not participate in as a result of their gender identification or sexuality. Overall, participants were involved in a large range of different sports. The most commonly reported sports that participants were currently involved in were soccer, Australian football, basketball, netball, and hockey. The most commonly reported sports played in the last 12 months were netball, softball, soccer, volleyball, and basketball.

**TABLE 1:** Summary of involvement in organized sport.

	Whole sample	LGBTIQ+	Hetero sexual
			
Variable	*N* (%)	*N* (%)	*N* (%)
Sport involvement	*N* = 145	*N* = 91	*N* = 52
Current	77 (53.1%)	49 (53.8%)	28 (53.8%)
Past 12 months	14 (9.7%)	9 (9.9%)	5 (9.6%)
More than 12 months ago	47 (32.4%)	28 (30.8%)	17 (32.7%)
Never	7 (4.8%)	5 (5.5%)	2 (3.8%)
*“Have you ever dropped out of an organized sport?”*	*N* = 137	*N* = 86	*N* = 50
Yes	93 (67.9%)	63 (73.3%)	29 (58.0%)
No	44 (32.1%)	23 (26.7%)	21 (42.0%)
“Are there any sport(s) that you have wanted to participate in but didn’t, as a result of your gender identification or sexuality?”	*N* = 147	*N* = 93	*N* = 53
Yes	35 (23.8%)	26 (28.0%)	9 (17.0%)
No	112 (76.2%)	67 (72.0%)	44 (83.0%)

**LGBTIQ+, lesbian, gay, bisexual, transgender, intersex, queer, and other*.*

#### Experiences in Sport

The survey asked participants to indicate a range of different experiences they had had in sport. Table 2 outlines the questions asked, and the level of agreement with each statement/question in LGBTIQ+ and heterosexual participants. The LGBTIQ+ participants reported experiencing a higher degree of challenges in sport in relation to their gender identification and sexual identity. More participants in the LGBTIQ+ community (39.7%) compared with heterosexual participants (15.7%) reported that they have felt unsafe or vulnerable in a sporting environment as a result of their gender identification or sexuality. Of those from the LGBTIQ+ community, this 39.7% comprised 9 gay, 6 bisexual, 6 lesbian, 5 pansexual, 2 queer, and 3 other. However, just over half of both LGBTIQ+ and heterosexual participants reported that they currently felt safe in their sporting club or organization, close to a third thought this was not relevant to them, and less than 4 and 10% of LGBTIQ+ and heterosexual participants, respectively, currently did not feel safe.

**TABLE 2:** Summary of experiences in sport.

	LGBTIQ+	Hetero sexual
		
Variable	*N* (%)	*N* (%)
*“Have you faced any challenges, issues or negative experiences in sport as a result of your gender identification or sexuality?”*	*N* = 81	*N* = 51
Yes	27 (33.3%)	8 (15.7%)
No	54 (66.7%)	43 (84.3%)
“Have you had any positive experiences in sport as a result of your gender identification or sexuality?”	*N* = 79	*N* = 51
Yes	41 (51.9%)	15 (29.4%)
No	38 (48.1%)	36 (70.6%)
“Have you ever felt unsafe/vulnerable in a sporting environment as a result of your gender identification or sexuality?”	*N* = 78	*N* = 51
Yes	31 (39.7%)	8 (15.7%)
No	47 (60.3%)	43 (84.3%)
Not relevant	0	0
“Do you feel safe in your sporting club/organization as a result of your gender identification or sexuality?”	*N* = 78	*N* = 51
Yes	44 (56.4%)	28 (54.9%)
No	3 (3.8%)	5 (9.8%)
Not relevant	31 (39.7%)	18 (35.3%)
“Do you feel included in your sporting club/*organization as a result of your gender identification or sexuality?”*	*N* = 77	*N* = 51
Yes	40 (51.9%)	32 (62.7%)
No	7 (9.1%)	3 (5.9%)
Not relevant	30 (39.0%)	16 (31.4%)
“Are there any policies in your sporting club/organization that you feel exclude members of the LGBTIQ+ community?”	*N* = 77	*N* = 50
Yes	3 (3.9%)	3 (6.0%)
No	19 (24.7%)	23 (46.0%)
I am not aware of my sporting club/organization policies	28 (36.4%)	11 (22.0%)
Not relevant	27 (35.1%)	13 (26.0%)
*“Are there any policies in your current sporting club/organization that you feel exclude heterosexual people?”*	*N* = 76	*N* = 49
Yes	2 (2.6%)	0
No	27 (35.5%)	28 (57.1%)
I am not aware of my sporting club/organization policies	22 (28.9%)	9 (18.4%)
Not relevant	25 (32.9%)	12 (24.5%)

**LGBTIQ+, lesbian, gay, bisexual, transgender, intersex, queer, and other*.*

#### Discrimination in Sport

Discrimination in sport was also examined. Of those within the LGBTIQ+ community, 79.7% had experienced (or witnessed) sexism in sport compared with 65.3% of the heterosexual participants. The majority of both LGBTIQ+ and heterosexual participants had experienced (or witnessed) verbal homophobia in sport (63.7 and 59.2%, respectively). Five percent (*n* = 4) of LGBTIQ+ and 2% (*n* = 1) of heterosexual participants had experienced (or witnessed) physical homophobic assault in sport.

#### Acceptance of LGBTIQ+ People in Sport

Lastly, participants rated their tolerance for a range of different statements related to the acceptance of different sexual orientations in sport. The items completed and responses are reported in Table 3. For the LGBTIQ+ community, over 88 percent of the participants were rated as being tolerant to all acceptance items in the questionnaire. For the heterosexual participants, the percentage of participants who were tolerant to each item ranged between 70 and 96 per cent. The items that rated the highest for ‘intolerant’ for the heterosexual participants were: “I would not like to get changed in the same changing room with a transgender person” (12.5%), “Gay sportspeople should not kiss each other and show off their homosexuality within public sporting events” (12.5%), and “I would never be part of a sports club that included homosexual, bisexual or transgender people” (12.2%).

**TABLE 3:** Summary of acceptance of LGBTIQ+ people in sport.

	LGBTIQ+^1^			Heterosexual^2^		
		
	Tolerant	Neutral	Intolerant	Tolerant	Neutral	Intolerant

Item	*N* (%)	*N* (%)	*N* (%)	*N* (%)	*N* (%)	*N* (%)
I would never be part of a sports club that included homosexual, bisexual or transgender people	68 (88.3%)	3 (3.9%)	6 (7.8%)	38 (77.6%)	5 (10.2%)	6 (12.2%)
If I were a coach, I would not feel comfortable knowing that there is a homosexual person on my team	73 (94.8%)	2 (2.6%)	2 (2.6%)	45 (91.8%)	2 (4.1%)	2 (4.1%)
If I had a child, I wouldn’t like their coach to be gay or lesbian	73 (94.8%)	2 (2.6%)	2 (2.6%)	41 (83.7%)	7 (14.3%)	1 (2.0%)
I would not feel comfortable hugging a homosexual rival after a match	70 (92.1%)	2 (2.6%)	4 (5.3%)	44 (89.8%)	4 (%)	1 (2.0%)
I would not feel at ease if it is known that my teammate is not heterosexual	74 (97.4%)	1 (1.3%)	1 (1.3%)	47 (95.9%)	2 (4.1%)	0
I would not like to get changed in the same changing room with a homosexual person	74 (97.4%)	2 (2.6%)	0	40 (81.6%)	6 (12.2%)	3 (6.1%)
I think that boys are not genetically suited for ‘artistic’ sports such as figure skating, rhythmic gymnastics or aerobics	72 (94.7%)	3 (3.9%)	1 (1.3%)	46 (93.9%)	2 (4.1%)	1 (2.0%)
If I had a son, I would not feel at ease if he wanted to practice rhythmic gymnastics or any other mostly ‘feminine’ sports	73 (96.1%)	2 (2.6%)	1 (1.3%)	42 (85.7%)	3 (6.1%)	4 (8.2%)
Girls who practice contact sports, like rugby, lose part of their femininity	74 (97.4%)	2 (2.6%)	0	46 (93.9%)	1 (2.0%)	2 (4.1%)
If a sportsman touches a teammate’s bottom when scoring a goal, it is because he is gay	74 (96.1%)	3 (3.9%)	0	46 (95.8%)	2 (4.2%)	0
If I had a daughter, I would not feel comfortable if she competed in rugby	71 (92.2%)	5 (6.5%)	1 (1.3%)	42 (87.5%)	2 (4.2%)	4 (8.3%)
Generally, I think that girls who practice sport are too muscular	72 (93.5%)	5 (6.5%)	0	46 (95.8%)	2 (4.2%)	0
I think that lesbians are more aggressive in sport than heterosexual women and girls	70 (92.1%)	4 (5.3%)	2 (2.6%)	40 (83.3%)	8 (16.7%)	0
It seems logical to me that sports fans would laugh at an effeminate player during a game	72 (94.7%)	4 (5.3%)	0	41 (85.4%)	6 (12.5%)	1 (2.1%)
I would not like to get changed in the same changing room with a transgender person	72 (94.7%)	3 (3.9%)	1 (1.3%)	39 (81.3%)	3 (6.3%)	6 (12.5%)
I would feel nervous in an openly homosexual sports club	73 (96.1%)	3 (3.9%)	0	40 (83.3%)	5 (10.4%)	3 (6.3%)
If I had children, I would not like their coach to be transgender	74 (97.4%)	1 (1.3%)	1 (1.3%)	39 (81.3%)	5 (10.4%)	4 (8.3%)
I think that most girls who practice my sport are lesbians	70 (92.1%)	5 (6.6%)	1 (1.3%)	41 (85.4%)	6 (12.5%)	1 (2.1%)
The image of lesbian sportswomen kissing each other to celebrate a victory should be avoided in sport events	70 (92.1%)	4 (5.3%)	2 (2.6%)	35 (72.9%)	9 (18.8%)	4 (8.3%)
Gay sportspeople should not kiss each other and show off their homosexuality within public sporting events	70 (92.1%)	5 (6.6%)	1 (1.3%)	34 (70.8%)	8 (16.7%)	6 (12.5%)

**LGBTIQ+, lesbian, gay, bisexual, transgender, intersex, queer, and other. ^1^Internal consistency (Cronbach’s α) for this sample was 0.94. ^2^Internal consistency (Cronbach’s α) for this sample was 0.95*.*

### Qualitative

Phase two of the research project involved collecting qualitative data. Participants who engaged in the phase one quantitative survey were invited to participate in the qualitative component as well. Sixty-seven of the participants who undertook the questionnaire provided their name and email for a voluntary follow up focus group or individual interview. Those who responded were booked into their preference of either a focus group or individual interview at a convenient location such as a University meeting room, cafe or local library. Thirty-one participants who had completed the online questionnaire participated in focus groups (*n* = 13) or individual interviews (*n* = 18). There were a range of key stakeholders (*n* = 12) such as coaches, executive staff, trainer’s, committee members, and officials. All key stakeholders were ex-sporting participants in their own rights. Twenty-four of the participants were still actively involved in sport. The other participants were ex-sporting participants (*n* = 7). Ages ranged from 16 years (with parental consent) to 62 years of age (mean = 31). Four participants did not disclose their age.

A semi-structured interview guide was utilized with questions that included:

(1) In what ways does your sporting club advocate for inclusion in sport?
(2) Who are the leaders of inclusion at your sporting club? How do they ‘lead’?
(3) What are your perceptions and experiences of homophobia in your sporting club?
(4) What lessons can sport learn about promoting safety and inclusion in sport?
(5) How does language and the sport environment impact how you experience sport?
(6) What improvements in attitudes, behaviors and/or policies have been made to include LGBTIQ+ persons in sport?
(7) In what ways does your sporting club accept or reject sexual diversity?
(8) In what ways does your sporting club accept or reject gender diversity?
(9) What opportunities can be seized by your club to promote safety and inclusion?
(10) What aspects of sport positively model inclusion and should be celebrated?

### Participants and Key Stakeholders

The break-down of participants in the focus groups and individual interviews were as follows:

- Of the 31 participants that took part in the interviews 24 were Adelaide metropolitan-based;
- There were 4 participants that were from Asian/Middle Eastern origins;
- There were 3 participants from rural and regional South Australia.

The intention of the qualitative component of the study was to capture both mainstream and niche sports. Table 4 illustrates the sport or recreational activities discussed, number of participants involved, including a both active participants and ex-participants. Table 5 displays the sports in which stakeholder participants are involved. It was essential to view both mainstream and niche sports experiences. These narratives comprise the richest qualitative dataset on sport and the LGBTIQ+ community to date in South Australia, and as such, offer naturalistic generalisability for the reader. Furthermore, while the number of participants captured within the qualitative component totaled 31, the data covers a wide variety of sports (*n* = 39) that are represented across the South Australian landscape. It could be argued that traditional sports may have historically imbedded gendered ideologies, particularly where masculinities and femininities are concerned. These can have significant influence upon constructions of perceived sexualities as well; including conforming to homogenous, heteronormative ideologies. However, there may be reason to hypothesize that newer, potentially niche, sports may not have to conform to traditional gender norms.

**TABLE 4:** Participant representative sports.

Sport/recreational activity	Active participants	Ex-participants	Total
Women’s soccer	4	3	7
Men’s soccer		4	4
Rugby (all codes) (women)	3	1	4
Rugby (all codes) (men)		1	1
Swimming		3	3
Dragon boating	3		3
Netball	2	7	9
Football	1	1	2
Gym	3		3
Softball	3		3
Basketball	2	4	6
Yoga	3		3
Water polo	1		1
Surf life saving	1		1
Hockey		1	1
Target shooting	1		1
Larping		1	1
Athletics	1		1
Medieval re-enactment	1		1
Dance (varying types)	3	1	4
Sailing		1	1
Boxing		1	1
Tennis		3	3
Table tennis		2	2
Frisbee (incl. ultimate frisbee)	2		2
Cricket		2	2
Volleyball		6	6
Badminton		1	1
Power lifting		1	1
Cycling	1	1	2
Running	2	1	3
Gaelic football		1	1
Lacrosse		1	1
Brazilian Jiu Jitsu	1		1
Martial arts		3	3
Jada		1	1
Baseball		1	1
Squash	1		1
Golf	1		1
Rowing		1	1

**TABLE 5:** Key stakeholders.

Sport/recreational activity	Total
Women’s soccer	1
Men’s soccer	2
Rugby (all codes) (women)	1
Swimming	1
Dragon boating	3
Netball	1
Football	1
Softball	1
Basketball	3
Water Polo	1
Badminton	1
Squash	1

### Qualitative Results

A number of key points developed from the data attained through extended questionnaire responses (*n* = 148), individual interviews (*n* = 18) and qualitative focus groups (*n* = 13). A thematic analysis was undertaken to carefully identify developing themes as well as the key issues identified by participants as being significant within their sporting community or organization. Some of these issues may be idiosyncratic to the sport or organization irrespective of their size or footprint on the South Australian sporting landscape. Nevertheless, the voices of these participants have been heard and need to be documented.

There were a range of themes that emerged in relation to a variety of topics including homophobia, sexism and gender discrimination, gender roles and gender stereotypes. There were also themes that related to the ways in which the participants perceived sporting clubs and organizations with respect to LGBTIQ+ issues and their role in changing short- and long-term behavior and cultural change. The themes that are presented below are based on this component.

### Theme: Homophobia

#### Homophobic Language, Attitudes and Behavior

Homophobic language has the capacity to impact members of the LGBTIQ+ community in many and varied ways. It is clear that in mainstream sports homophobic language continues to be used frequently. Participants in this research identified the following homophobic terms that they hear on a regular basis during sport participation: ‘poof’; ‘poofters’; ‘dyke’; ‘gay’; ‘fairies’; ‘fag’. However, they did claim that this language might not always be used in a literal manner and intent behind the use of these terms. Regardless the homophobic language was used in a derogatory way to offend and emotionally challenge opposition players.

*Well, I think language impacts your experience of everything. And yeah, in terms of that sledging that did, I think had we not been sort of a supporting and fairly large team I think that would have seen us out of the competition for those experiences. Because I do remember sort of a palpable and sort of tangible feeling of bracing ourselves for playing against certain teams in that competition because we knew it would happen. And you know in volleyball where you have like the service rotation you know, when you’re up to serve, you know, you’d have everybody on the opposing team watching you for sporting reasons, but that’s when it would happen. And you just sort of line up, you’d go and put your foot on that line and go to serve and just, firstly hope you did a good serve because otherwise if you didn’t the reason would be that, you know, the reason would be given a sexual connotation, or it would be gendered, or you know that would be why you’re accused on not performing.*

Significantly a number of females in this research, who identified as gay, were adamant that the word lesbian has severe stigma attached to it. They discussed how confronting it is to be called a lesbian. Indeed, one participant specifically claimed to have a physical reaction to the word lesbian, “It makes me feel physically sick.” This is a very important issue that has been raised by these participants who are a part of the LGBTIQ+ community particularly with the way in which the term lesbian creates feelings of disquiet amongst gay women.

*Even if you’re a woman that is really good at football, you’re probably going to be called a dyke or something like that, you’re just a lesbian, sort of thing, that’s why you’re good.*

And:

*And also, like, on the opposition maybe, it was used as an insult, less within the team but if an opposition had players which were perceived as gay or manly, or something like that, they would sometimes be derived, derided, like insulted using the gay or insults like that.*

Similarly:

*Like there’s a bit of shame associated with the fact that, oh everyone who plays this sport is gay, and like, with the Gaelic team, I never felt being part of the team. I felt really comfortable and accepted and stuff, but when I tell other people that I play Gaelic football, I always feel the negative connotations of it and slightly self-conscious about it.*

It can be argued that homophobic language is a form of homophobic attitudes and behavior. However, within the context of this research homophobic language was generally confined to sporting competitions as a form of vilification or inadvertent derogatory challenges. In terms of physical behavioral acts participants had not necessarily experienced specific homophobia. They did claim that there were occasions when specific targeted harassment would take place. Indeed, it was suggested by a number of participants that once there is exposure of sexual or gender diversity within the club, team or organization, there is potential for people to be treated differently. As one participant identified:

*Q: So, there are certain stereotypes then that sort of come into it?*

*A: Definitely stereotypes, yep, and that’s where I have a giggle because I think, well I’m one but I’m not this big, scary player that’s – they think that you’re dirty and you’re going to foul each other off and act really badly just because you’re a lesbian, it’s actually quite funny.*

*Q: Does anyone ever say anything in relation to that? Have you ever said anything or anyone else in the team ever sort of reacted to someone who has made these sorts of comments?*

*A: No, it’s more they all just laugh and agree with each other and I guess me being a minority and I guess being new to the club and new to the team, I sort of didn’t feel comfortable in saying, ‘hang on a minute, you’re sort of talking about me when you’re talking about me.’ Yeah, I didn’t feel comfortable enough to say that.*

With respect to homophobic attitudes, discussion regularly developed around the supposed ways in which lesbians speak and dress, or their perceived level of threat based on sexuality. There were also commonly held beliefs that many straight people within sporting clubs and organizations viewed males together (i.e., gay men) as ‘disgusting.’

*If the Jiu Jitsu club was majority homosexual – openly homosexual men – I’m not sure that I’d want to go there.*

A participant also recounted an example of homophobia that occurred within women’s basketball. It was claimed that the dynamics completely changed as a consequence of a suspected lesbian on another team. This created what the participant described, as a ‘threat’ as teammates irrationally perceived that the lesbian had a crush on all the women in the team. Therefore, as a result of these irrational assumptions there was a good deal of discussion, homophobic language and differential treatment toward this woman as a consequence of her sexual diversity.

*A: I reckon and I was playing hockey with a group of girls who I had been through school with, most of them, we’d been mates forever so really good friends and easy going with each other and we played against this team that there was a woman on the other side that was a suspected lesbian, nobody knew for sure but she was suspected so the – most Saturdays was talking about her, whether she was or wasn’t and her behavior on the field, whether she, I don’t know, looked at someone or whether she – at the end of the game you always shook hands so it was all like, oh my God, she shook my hand and she held on to it a bit longer and obviously she thinks I’m alright and, I guess, just all that. That’s probably my first memory of it, definitely. This poor woman, whether she was or wasn’t, she was labeled and then just treated differently because she may have been and also the women felt really threatened because they were thinking that maybe this woman might have had a crush on them and they were just like, oh gosh, what would you do? It was really interesting, the dynamics how it changed, just one woman on the field. It was only suspected.*

*Q: And then the whole sport became about her as well.*

*A: Yeah, yep, definitely. So that’s probably my first one. The rest has all just been when they’re just in groups and they are always talking about homophobia and always talking about lesbians and always talking about how bad it is and even when they start talking about the men and calling them poofters and gay and it’s always a detrimental thing and, yeah, so probably just more that sort of speak, that really homophobic speak around it.*

*Q: And always in that negative, sort of derogatory way?*

*A: Definitely derogatory and really threatened by it or them, I think some of them would probably actually be insulted if they thought this woman liked them and then she didn’t, it sort of insults them because they’re like, well obviously lesbians like every woman they see, so you’re quite insulted if they don’t like you.*

Homophobic language, attitudes and behavior are still prevalent, and in existence, despite the argument to suggest that that has been a decrease of occurrence across generations (Denison and Kitchen, 2015). The participants in this research were adamant that no form of homophobia should exist in general society or in sports. Given that some of the participants stated that targeted harassment can occur in sports, and a small proportion had experienced homophobic behavior and attitudes from others, support is required to immediately arrest all forms of homophobia.

### Theme: Generational Shift

There was recognition that society as a whole has shifted its views related to the LGBTIQ+ community. There were numerous comparisons to previous generations and how society, including the sporting sector, has become more accepting and inclusive toward the LGBTIQ+ community. This includes that people can be more open with their disclosing their sexuality and introducing their same-sex partners. In past generations it was argued there was an intense fear based on prejudice surrounding the need to hide one’s sexuality. This was stated to be both society-based, and in sports clubs, teams and organizations. It was also noted that from the early 2000s, there has been a shift away from stereotypical male and female media representation, where there was little related to anything but the heteronormative image. The participants claim that there has been a shift within current media representation.

*I don’t know what’s driven it, but there is definitely a big change in how many sports people are out. There’s probably not as many men as there are women I don’t think, again not citing hard data on any of this, but like, with basketball because that was then only one that I followed, I did notice there was a year when I was injured*…*Yeah so I did all this tracking, anyway back to the original story, I did all this tracking and I collected a lot of data and I noticed that in the WNBA because that was the league that I was following, that was the league that was the league that was playing at the time and it was the league at that year there was 16 Australian players in it, which was massive. I noticed that there were women that would be interviewed about their family and they would appear, these players, they would appear in photo shoots with husbands and children, and it would all be this whole family thing. And then there were other players where, who would never be, well that information would never be published, it was just like a no comment zone. And I sort of started to think well you know that’s obviously because they represent sort of a non-normative sexual identity. But I’ve noticed that that’s not so much a thing anymore. I actually did a whole stack of digging around because I thought ‘well is there some kind of specific team policy that’s preventing these women from talking. Is it their own personal fears or hang-ups or just their own concern for their career that is stopping them from being interviewed or is it a lack of interest from the media’? I don’t think it was a lack of interest from the media I think that probably would have been created quite a bit of interest. But that was, it was really actively excluded from the discourse around women’s sport at that time 2001, 2002. But that’s definitely changed and to a point obviously, I don’t think we can mark that one down to fixed, but that’s really changing, you do see like the Erin Phillips basketballer, she was on the front page of the Advertiser with her partner and their children, and she’s received a lot of support in that. And the image around her, like the public imagine around her is overwhelmingly positive.*

It was noted by majority participants that as a society, we are less label-oriented in terms of having to identify an individual’s particular sexuality. It was also argued that we tend to care much less about this than previous generations. This could be in part due to the greater level of social conscience that is held by younger people. Therefore, instances of discriminatory behavior would not be tolerated, comparatively to previous generations.

*Q: So, from that example, it still sounds like sexuality or sexual orientation can act as a barrier to participation in sport.*

*A: Oh, absolutely. And I guess the AFLW has done a fair bit of work on that, and again, the marriage equality campaign where you saw sports come out and support that, making statements in relation to that, and not just sports, but businesses and government departments where they actually made a declaration, well this is about all of our people not just a part of our people, and we should be advocating for all of our employees not just a percentage that don’t agree or have some kind of challenge with it, because at the end of the day, that’s their challenge, not ours, that’s the way I look at it. But yeah, I just, I think if people were a little bit more aware and educated about it, then you probably wouldn’t see – you’ll still see some challenge but not what we’re getting at the moment.*

With respect to sporting clubs, which are often historically imbedded with a member’s culture and “way of being,” there can remain a fair degree of resistance from older generations toward such aspects as diverse genders and sexualities. Specifically, this ideological resistance can emerge from committee and board members. Clearly this can be problematic with respect to creating an inclusive agenda and, considering the importance that all participants in this study stated, in having a ‘top-down’ approach. It was argued that without a holistic approach by the club or organization there needed to be significant change in terms of ousting the “old guard” from positions of power such as committee and board membership. This includes the presidents and chairs. They further claimed that this was one way in which there could be true systemic and long-term ideological and cultural change. These are powerful perspectives from the participants which is reflected by the following comment:

*It starts right at the top with governance with the culture at the top, so setting an accepting culture of all people, and actually walking the talk in relation to that and not just it being these are our values and we’re saying we’re inclusive, but we’re not actually being inclusive. So, it starts with all the policies and all the governance documents, but it’s also then about creating really good role models in relation to that. And some people find that difficult to embrace, but you know, making sure that people understand that it’s not a barrier, it’s not a challenge, you can be a senior exec, you can participate in sport, you can be an official, you can be whatever it is, a volunteer, and it doesn’t matter, you’re just a volunteer or whatever, rather than all these other names that you’re allocated. So, I think, and then generally, working right down with your associations. Because often people are scared of what they don’t know or don’t know how to create an environment of inclusiveness, so rather than try to do it, they’ll just not do it. So, I think making it okay for people to participate and to volunteer and to manage, really. But it’s got to be incorporated in everything that you do, and it can’t be what I call a bolt-on, so you know, this is for this community, and this is for – no, it has to be part of everything you do. It’s the same as a gender diverse argument, it is no different. So, there are some really good examples around gender diversity and how they’ve created that in terms of the – not the me-too campaign, that’s to do with sexual harassment, but I can play too, or their campaigns that they’ve got now in relation to women getting physically active – it’s no different. We just need to be seeing those on our screen more regularly, and hearing about them more regularly on radio and out in the community, you know, without pointing to their sexual orientation or anything else.*

It was recognized that latent homophobia still exists in sporting clubs and that attitudinal changes are slow, as previously identified. It was argued that much of the attitude and behavioral change was created as a consequence of the broader social and cultural changing laws, such as those associated with marriage equality and same sex relationships, as well as recognizing the rights LGBTIQ+ community are equally valid in society as they in sporting clubs and organizations. Similarly decreasing the level of stigma associated with the LGBTIQ+ community in sporting clubs and organizations is pivotal to creating long-term positive systemic change.

*Well definitely, definitely and I think that’s, I think that’s indicative of a generational change, there was a really, I don’t know if you read it there was a really interesting piece in the conversation that looked at how the, I suppose accepted generations that we talk about, like the millennial and the X’s and the Y’s and baby boomers, and how that aligns with perceptions around sexual identity and it, I just found that that really sort of rang true. There was a, the generation before me which were really active campaigners or not, who had a certain view around things and then there was my generation and then they talked about a generation, so if you grew up in the 70s or 80s there was a particular name for it, I can’t remember what it was but if you grew, if most of your childhood was in the 90s or afterward they called it a post gay generation where an attitude, it was in the conversation it was really good I’ll send it to you. Where attitudes really shifted and that’s the generation that we’re seeing being really active now, and I think that’s driving that kind of change. I think it’s; I think they called it post gay because it was, yeah, it’s a generation where labels don’t matter as much where there’s a great deal more fluidity in by terms of sexual identity and gender.*

### Theme: Sporting Organizations and Clubs

#### Leaders/Governance

There was overwhelming commentary from the participants in this research claiming that positive, welcoming, inclusive governance from sporting boards and committees, as well as other significant leaders, is integral to club’s being inclusive to the LGBTIQ+ community. Leaders can include board/committee members, coaches, team managers, captains, and individuals that exist in teams as a consequence of longevity and informal social structures. It was recognized that anyone within the club or organization has the capacity to be champions of inclusion. However, when participants were asked about whom the leaders of inclusion in their respective clubs were, it was generally staff or volunteers with official titles who were quickly identified.

It seems the process of governance together with leaders, and champions of change, are the most significant factor in determining whether the clubs are likely to be inclusive, according to the participants in this research. There was significant discussion surrounding the notion that inclusivity must be driven from a “top down approach” meaning that leaders, such as boards, committees, presidents, CEOs, coaches and senior players need to adopt attitudes and behaviors that are intrinsically inclusive. While participants were clear that inclusivity from leadership is fundamental for clubs to be welcoming and inclusive, this same approach must be adopted and promoted by the overarching sporting associations. The participants further claimed that leadership in the highest forms, such as the CEO of a sporting organization must be inclusive; otherwise there are serious challenges that exist to create systemic change amongst clubs at the “grassroots.” Without a broader inclusive framework from the governing body to assist the clubs on adopting change policies the clubs will struggle to develop their own set of strategies. The following comment is typical of the responses that were stated throughout the research.

*So, I think something needs to come from the top level of those sporting associations to absolutely condemn that kind of behavior and make a really, really clear assertion that there’s no place for it in sport.*

It was also recognized that older players, senior role models and mentors within clubs are crucial toward developing an inclusive environment and enacting inclusive practices that are visible to other team members. This includes attitudes, behaviors and language both on and off the competitive sport setting. Much of what can be learned within the club environment takes place in locker rooms, at training and in social settings. This is an approach that the participants have identified as “leading by example.” However, in theoretical terms it is the essence of social constructionism whereby younger players learn a way to “act” and understand what is socially, morally and ethically appropriate through sound leadership within their peer group. One of the participants reflected this by claiming:

*I think it needs to be made very clear what constitutes inclusion or what doesn’t, what constitutes an appropriate sporting culture and what doesn’t, and I think that needs to be monitored and enforced and for there to be a zero tolerance in a style of leadership.*

Often there is a hierarchy within teams based on skill level, and those who are the most skilful participants are at the top of this hierarchy. These leaders must be inclusive, or teams can become exclusive very quickly. It is incumbent upon the club to assist in the development of educating these senior players about inclusive practice. Simply being a good player does not provide the skills and abilities to be a good mentor or role model. It is also important for the coach to be an integral part of the inclusive narrative and practice within the club given that coaches are seen as the “next layer” following on from senior peers within the team. Participants experienced both positive and negative coaches, who are influential leaders in their own right. It seems the coaches play a pivotal role in being the conduit between the governance structure of the board and the team, senior role models and mentors, and individual players. One participant stated that they had never experienced a positive coaching leader. This participant claimed that; coaches need to set the example of what is “expected behavior.” He also argued that leaders, including coaches, sometimes demonstrate inappropriate behavior, and ‘will not listen’ or are ‘ignorant.’

*Q: Okay, and in your own sporting club who are the leaders of inclusion would you say? Who really encourages that, that culture?*

*A: More or less everyone. The current club leader has, oh he’s another crossbow shooter. He’s been with the club for a long time and he helps pretty much everyone out. He doesn’t really care what you are, who you are as long as you ask politely, he’s probably going to be able to help you out with just about everything you ask. The other main member of the crossbow section of the club I suppose would be Liz Johnson. She’s pretty much one of the best in the world. She has competed at a world level multiple times and she’s just happy to get in more people that are shooting crossbow in general. Again, she doesn’t really mind who it is, but she’s been a very, very fun person to work with and learn from.*

Women specifically stated the importance of having same-sex roles models and coaches within their teams and clubs. Exposure to leaders such as this provides tangible evidence that women can assume significant leadership positions within the club. Additionally, it was claimed that the most inclusive practices that many of the female participants had experienced were associated with female coaches. For female participants who identified as a part of the LGBTIQ+ community, having an openly gay or lesbian female coach was of great benefit to their inclusive sporting experience. Noteworthy many of the female participants, irrespective of sexuality, stated that inexperienced male coaches, of female teams, tend to be ‘scared’ and treat them with ‘softness’. They also claimed that they could be somewhat oblivious to what is going on in the team given their lack of understanding of women. While participants do not necessarily perceive that coaches need to be heavily involved in the team dynamics beyond the competitive element, having an awareness of players’ sexuality is an important aspect they should know and understand. There is often a clandestine association between male coaches and the sexuality of female players. One participant specifically stated that they have been treated differently (positively) now their sexuality was exposed to their male coach. It was identified there is a strong need for more female sports coaches and increased female membership on boards and committee members. The importance of diverse representation in all facets on boards and committees was illustrated. Therefore, the need for more women “at the top” was a clear message being sent for the participants.

*Yeah, and it’s typical of society, right? A whole group of women being controlled by a man. Like, why don’t we have female coaches? Like, in the teams where I’ve had female coaches, we’ve been much more successful because they understand how the game is played differently, and they understand how motivation is different between the genders. And it’s true that, like there is, you know there are subtle differences, and it’s just that feeling of the coach being part of the team. I’ve only ever experienced that with female coaches. With male coaches it always seems like there’s something separate, and I guess that keys into like hiding the sexuality and stuff, they don’t feel part of it, whereas with female coaches I haven’t had that problem.*

There was also little doubt from participants in voicing their opinion that some board members of sporting clubs and organizations need “to move on.” These board members were often seen as older, white males, who are “stuck in their ways,” thereby making change difficult to implement. They also suggested that without “moving on” many policies are often out-dated and not necessarily inclusive of diverse genders and sexualities.

*I think there needs to be really clear policies around it. I think there needs to be really clear ramifications for violations of those policies. I think it needs to be made very clear what constitutes inclusion or what doesn’t, what constitutes an appropriate sporting culture and what doesn’t, and I think that needs to be monitored and enforced and for there to be a zero tolerance in a style of leadership.*

Another participant claimed:

*And it’s usually dependant on the hierarchy of the association that you’re with. If it tends to be a younger age bracket they will be more inclusive of everyone, if it tends to be the old guard still running the show, then they will put in the rules but they’ll be out aside to hide In the corner sort of thing, and there is nothing that you’re able to do until you can change the old guard. Your separate clubs will be able to fix it because they will tend to be the younger ones, but the overarching association will hide it away. So, it’s probably what you classify as active discrimination, but it’s by the old guard not by the young ones.*

There was recognition by the participants that further education is required for boards, committee members and coaches regarding inclusivity to the LGBTIQ+ community and the use of appropriate language. The emerging sport of LARPing (Live Action Role Playing) was cited as good example of inclusive practice and policy development. It was claimed that policies and procedures were specific to gender, and sexuality diversity and a comprehensive member protection policy exists. There were several leaders, all of whom are inclusive, welcoming and communicate with participants. The board are regarded as friendly and welcoming, and there is a specific, trained, liaison officer to consult in times of concern. Additionally, a briefing on respectful behavior is conducted at the beginning of each event to remind players and spectators of the underpinning cultural values of the sport.

*They just have a brief at the beginning of events where they’re, no, don’t be an idiot to people thing and make sure that you’re not just, they just try and make sure that people have read the rules they’ve written thing, make, they’ve got it on their Facebook group. And it’s, it’s a thing when you sign the membership form thing you have to say, I’ve read, I’ve read all of your terms and it’s, it’s in that so that’s cool.*

#### Policies

Policies are crucial pieces of legislation that can have an enormous influence on the culture of a club, team or organization. However, for many participants policies were somewhat non-existent or were merely a part of the burgeoning bureaucratic landscape. It seems that unless a participant was involved at the committee or board level of a club, then they were oblivious to many of the club’s policies and procedures, specifically related to inclusion. However, some participants had a vague awareness that there would be something within their policies and procedures to ensure that discriminatory behavior would be dealt with in their club. They were unsure of the specifics and had little idea as to the ramifications in the event of discriminatory behavior taking place. As one participant stated:

*The policy is pretty vague, but as a statement I don’t think there’s anything either way to say we’re inclusive or we’re not inclusive, it just is what it is.*

There can be a discrepancy between policies and the behavior of the organization/club. Apart from unambiguously discriminatory legislation, participants stated that there were no explicit policies and procedures related to gender and sexuality. LARPing was the only sport that had gender and sexuality specific policies and procedures. A number of participants stated that there are no specific policies related to transgender people, which is clearly an area that requires further investigation. In the absence of policies, it was suggested that clubs might sometimes use the policies of bodies such as Sport Australia as encompassing “catch all” guidelines in the event of clubs being unsure as to what they need to include.

Policies and procedures are integral to creating a club that is welcoming and inclusive. They can also be educative on sporting participants’ requirements. Overarching policies and procedures are required in order to eliminate the potential for discrimination to occur as well as provide procedural recourse in the event that incident does arise. Many of the participants in this research claimed that, in an ideal world, all members would need to be aware of club policies and procedures. Any discriminatory behavior, including the use of homophobic language should be sanctioned, with clear step-by-step processes, including those responsible for “ongoing follow up.”

One of the policy issues that emerged within the research was that associated with transgender athletes. There was significant debate among the participants about rules, regulations and laws that are associated with transgender athletes and their sporting participation. It is clear that transgender people must deal with a range of issues beyond those of lesbian, gay and bisexual individuals and therefore, within a sporting context, a number of the participants identified that sporting clubs and organizations are “just not well set up to cater for transgender participants” within their club or organization. This includes infrastructure in terms of having adequate change room facilities to accommodate for a variety of sexualities, nor the education and training, which is critical in moving forward. For example, a swimming coach stated that, “I don’t feel we have the tools including education or expertise on how to help the sporting participants who may identify as transgender.” While there was a discourse surrounding facilities, including change rooms, as mentioned, for transgender people, there was a perception of transphobia related to change-room access, which needed to be dealt with at the policy level.

*A: Yeah, I mean policies and procedures – what do they do about somebody who comes and says they’re transgender? – they understand that what the problem is which changing room do you go and get changed in. It’s a really basic sort of problem to have clear guidelines about.*… *and understand what the issues are and the programs I guess.*… *and make sure they understand what they want to be called.*

Arguably the major factor that needs to be taken into consideration with respect to transgender athletes within sporting clubs and organization is the lack of consultation that has occurred and the need to increase the level of awareness and education that surrounds this important aspect. According to participants the lack of education and development of policy is a significant area of concern that needs to be addressed immediately within the sporting landscape.

*And one of the ones that kind of more difficult at the moment is the transgender policy, and I feel they kind of got left out a little bit, and you know, we talk about marriage equality and we didn’t really talk about transgender. We didn’t really give them a platform to talk about their level of discrimination, which I would imagine, because I’m not transgender I don’t definitely know, but I would imagine it was much, much worse than the discrimination I’ve experienced. But so, I’m just not aware of any policies. And yeah, look there might be some policies, but I’m not aware of them. It’s alright for the Office for Recreation and Sport, or other state bodies, to say you have to provide a welcoming environment and not be discriminatory against anybody, because that’s the legislation, but for the people on the ground in club land and association land, what does that mean?, you know, and how do they do that, and how do they deal with their biases. So, no, I’m not aware of any legislation in South Australia.*

#### Environment, Resources and Facilities

A number of the participants identified the “environment” of the club as being important to the overall “feel” as to whether it was welcoming, inviting and inclusive. Environment can mean a variety of things to different people. It may include the physical, social or emotional space. Clearly a range of factors plays into the notion of environment. The creation of policies and procedures is one aspect of creating a positive social and emotional environment. However, the physical environment also needs to be taken into consideration. Some of the women mentioned the way in which the majority of sports are highly masculinised environments and seem to largely cater for males. Many examples were provided around masculinised spaces such as the weights rooms in gyms and fitness centers. This made a number of women, irrespective of sexuality, very uncomfortable. One woman stated she was “terrified” to enter the weights area of a gym for fear of the large muscular men “invading” the space.

Girls and women involved in this research identified that their needs including resources, training allocation, space and, at the elite level, pay disparity, are secondary to males. There were examples of male soccer coaches encroaching on females’ soccer space at training thereby limiting the women to a smaller playing area. There were also claims of women having to use second hand equipment while males used the newly purchased items. Some participants stated that males simply had a greater a voice within sporting clubs, displaying a lack of respect for women in the club. This inequity does not inspire confidence in females participating in these sports. The comparison between resources to sports at a Government level was also provided, with claims that larger, more powerful, sports receive substantially more resources than niche sports, making it challenging for increased participation and retention in these sports.

Resources are required for clubs, leaders, specifically coaches, parents and members which detail evidence-based acceptable and unacceptable behaviors, language and examples of the way in which inclusive clubs appear. Easily accessible instructions and guidelines would assist clubs, especially since coaches are generally under-resourced already. Importantly this is not simply about women and the sporting environment in which they exist. Gay men involved in highly masculinised sports also struggle to come to terms with the traditional hegemonic masculine space created within these domains. While the statistics indicate there are no gay men involved in elite level football (all codes) in Australia there are likely to be gay men involved. Similarly, there are likely to be gay men involved in a variety of other traditional masculinised sports across all levels of sport in Australia. These environments make it incredibly difficult for men to divulge their sexuality to teammates for fear of reprisal and retribution. As a consequence of such retribution they may not feel like they want to play any longer. Therefore, in order to remain in the team is easier not to “come out” to teammates and simply play while masking one’s sexuality.

### Theme: Intrinsic Inclusion

One of the dominant themes to emerge was that of intrinsic inclusion within the sporting and club environment. Using the term “intrinsic inclusion” means that the majority of the LGBTIQ+ participants in this research wanted to play their sport and not be recognized as a LGBTIQ+ individual playing sport. They simply wanted to be another team member. The following quote form a participant is representative of the claim that has just been made and is representative of numerous comments made by participants:

*I guess, for me, because I was in the closet or kept it hidden for so long, for me it’s, I guess I don’t like to announce it and I don’t like it in those sort of circles because I’m a really private person because of that, to me it’s just, to me it’s, to not talk about it and just, I don’t know, accept people for who they are and not have to advertise it, not have to have, what would you call it, meetings or all these things about inclusion because then that’s – that’s what separates you, do you know what I mean? It’s when you don’t have to talk about it, but people just rock up to play.*

Similarly, another man stated:

*To me inclusion is we all just talk about each other and to each other and play the game and it really doesn’t matter who you go home with at night, who you’re living with or who you choose to love.*

It was identified that individual sports appear to be far more “safer” for younger athletes who identify as LGBTIQ+. For example, swimming was seen by many as a sport that was inclusive of genders, sexualities and disabilities. The sport has a long history of inclusive practice and has an enormous presence within Australia as a leading sport underpinned by a significant national organization in Swimming Australia. It was claimed that, “swimming is more equal than lots of other sports. It’s more female friendly as well.” Participants also recognized individual athletes as having the opportunity to “mask” their sexuality in a far more controlled manner than in team sport environments. Further it was argued that team sports can often be quite “random” and those with louder voices and personalities can influence the overall environment irrespective of the policies and procedures that are put in place. However, where gay and lesbian women are concerned, there are certainly some sports, as identified earlier, that are more inclusive than others, such as soccer. The problem, however, is the public perception of such sports being emblematic of gay and lesbian women and therefore risk the potential of straight women gravitating away from the sport for of being label gay or lesbian.

Once again, irrespective of sexuality there are major hurdles for sport to develop inclusive practices and policies given the gender divide in which we exist. The historical, social and cultural barriers within sports mean that it will take time to create systemic change. This was noted by the participants within this research as one man claimed:

*It’s going to be a big task for inclusion, because you’ve got to get past the gendered side of things, so boys play footy and girls play netball or girls play netball, not boys. So, yeah, it’s going to be a long road.*

## Discussion

As it has been identified throughout the data the paramount importance for policy and practice surrounding inclusivity where the engagement of the LGBTIQ+ community in sport is concerned. Both insiders (i.e., athletes, coaches board, and committee members etc.) and outsiders (observers) agree that this is crucial for change in this area. The problem is that clubs are struggling to create strategies with respect to the way in which they develop these policies and then implement the practices.

Many of the clubs are essentially run by volunteers often with little or no formal education, knowledge and understanding around issues associated with inclusivity where gender and sexual diversity is concerned. It is not uncommon for clubs to be almost 100% reliant on volunteers to keep the club functioning. This includes coaching, committee membership, fund raising and transport to name few areas. These volunteers come from “all walks of life” and are more often than not related to one of the players whether those players are their son, daughter, partner, mother, father, brother or sister. These volunteers may not necessarily be academically educated or maintain a vast range of cultural understanding in relation to changing sociocultural ideologies where sexually and diversity is concerned. Some may be opposed to this ideology; others may be champions for the cause, while others simply may not care. However, this does not make them less valuable to the club, as most volunteers are doing what they perceive to be the best thing for the organization. Clearly education is the key, which is “easier said than done.” The need to develop education tools is incumbent upon the governing bodies for the clubs to implement in the best way they see fit. How this is done is a significant question that requires further consultation. Another significant question is who pays for this education and when and how do the volunteers fit it in to their lives?

One of the other issues that can impact the development and implementation of policy and practice is the competing agendas that exist within a club. For example, a primary aim of the coach is to win games. While creating a club culture that positively values sexual diversity and inclusivity could be regarded as being important, it is generally secondary to winning as success is often seen to create a good culture. Coaches often use the mantra of “success breeds success” and “success creates a happy environment.”While this is certainly possible it is not necessarily the case with respect to developing a long-term culture underpinned by sound values, principles and practices.

A similar claim can be made about boards and committees where winning and success is concerned. While the club and coach at the “grassroots” level may be seeking change and attempting to develop long-term ideological change the board and committee may be seeking immediate success based on a number of competing agendas. For example, the board may be attempting to appease sponsors by attaining success. It is not uncommon for sponsors to place a win/loss ratio clause on sponsorship deals together with potential for sponsorship extensions based on this ratio. Ultimately, the actions placed on attempting to win are often financially driven. Therefore, while the club may espouse the virtues of being inclusive and identify the importance of developing policy and practice in the area, short-term winning and immediate success can take precedence at the expense of this development.

As it has been identified throughout this paper a key component to inclusive policy and practice within sporting clubs and organizations is education. Irrespective of the sport, the sexual or gender diversity, the locality, or the level at which the sport is played (i.e., elite vs. community) education across all domains is paramount. Education from the “top down” is regarded as arguably the most considered and resourceful approach given that the head of the club or organization is providing an endorsement for education around these key issues to occur throughout the entire structure. Having a “champion of change” through education at the helm of the organization means there is a greater chance of systemic cultural change.

The next level of change through education that is required must occur at the President/board/committee level. Endorsement and action through policy making is imperative at this level. It is where unity and solidarity are forged in order to provide a clear message with respect to what the club stands for. Once again, not all board and committee members may be well versed in matters pertaining to gender and sexual diversity and inclusivity within a sporting context. However, as a collective it will be incumbent upon this group to become educated in this area in order to create long-term change. This will need to be built into policy for future board and committee members as they change over time.

There are a range of coaches that exist within sporting clubs and organizations. Some are full-time paid coaches of elite and pre-elite squads and teams. Others are part-time coaches of emerging athletes while others are simply volunteers doing the best they can under the circumstances. Given that the coaches are at the “coal face” and in contact with the players and athletes the most they are likely to be the person responsible for delivering the inclusive message to players and being the “face” of the club to supporters and stakeholders, including the board and sponsors. Therefore, if inclusivity is to be adopted, maintained and perpetuated as part of a larger initiative, it is the coach that needs to be aware and selling the message to his or her athletes. Similar to committee members, some of the coaches that take on these roles have little or no education in the field of gender and sexual inclusivity. It is arguable that, it is up to the committee to provide the resources and support to enable education to occur.

Finally, the supporters of the team play a significant role in the culture of the club. Having educated supporters around the issues associated with gender and sexual diversity makes the environment of the team feel more inclusive, and there is potential for their attitudes, language and behavior to be a positive educative tool for opposition supporters as well. Supporter based education is more likely to emerge from the way in the coach; the team and the organization present a constant and consistent message around inclusivity at the expense of everything else. Changing a culture of a club takes time and the supporters need to be aware of the path in which the club is heading.

The issues surrounding inclusive practices among the LGBTIQ+ community and sport is incredibly convoluted, confusing, challenging and dynamic. These are a just a few terms that can be used to describe the environment in which the LGBTIQ+ community exist with regards to sport, and seemingly within many other areas of life. The qualitative and quantitative data within this research has highlighted a number of issues that confront the LGBTIQ+ community where sport is concerned.

While the research data is rich and compelling, there was significant difficulty in attaining quantitative research responses in association with the online questionnaire. There could be a range of reasons for this given that increasingly diverse and marginalized groups are invited to complete questionnaires, which could be contributing to research burden. Conversely, the research team had a number of participants seeking to be involved in the qualitative component to allow their voices to be heard. This is a very important research outcome in itself. It is clear that members of the LGBTIQ+ community want their stories told and their voices to be heard in relation to sport.

## Data Availability Statement

The raw data supporting the conclusions of this article will be made available by the authors, without undue reservation.

## Ethics Statement

The studies involving human participants were reviewed and approved by Flinders University Social and Behavioural Ethics Committee. The patients/participants provided their written informed consent to participate in this study.

## Author Contributions

MD is the lead author and lead researcher. SE and CD assisted in qualitative research. IP and LL assisted with the statistics which underpin this research. NB collected the data. All authors contributed to the article and approved the submitted version.

## Conflict of Interest

The authors declare that the research was conducted in the absence of any commercial or financial relationships that could be construed as a potential conflict of interest.
